# Enterobacteria Survival, Percolation, and Leaching on Soil Fertilized with Swine Manure

**DOI:** 10.3390/ijerph20075283

**Published:** 2023-03-28

**Authors:** William Michelon, Nivia Rosana Weber Peter, Tamili Martins Schneider, Dara Cristina Segalla, Aline Viancelli

**Affiliations:** Universidade do Contestado, Rua Victor Sopelsa, 3000, Concórdia 89711-330, SC, Brazil

**Keywords:** land application, pathogens, *E. coli*, *Salmonella*, clayey soil

## Abstract

Swine manure has a high load of pathogens, which can pose a risk to human and environmental health. In Brazil, studies evaluating the survival of pathogens in soil are scarce. Therefore, this study aimed to evaluate the survival, percolation, and leaching of enterobacteria in clayey soil after fertilization with swine manure. For this purpose, soil columns were fertilized with manure spiked with enterobacteria. The microorganisms’ behavior was monitored in terms of survival, percolation, and leaching with and without rain. Soil samples were collected, and Escherichia coli and *Salmonella enterica* serovar Senftemberg were quantified. The results indicated that *E. coli* survived for a longer period (43 days) than *S. senftemberg* (14 days). *E. coli* percolated quickly through the soil, leaching 60 cm in less than 5 min during rainy events and remaining viable for up to 24 h after the rain. The results show the importance of treating manure effectively before being added to the soil. An efficient treatment could be anaerobic digestion, followed by a pond system. Considering the characteristics of swine-producing regions, the load of effluents applied to the soil may percolate, leach, or run off and consequently contaminate water bodies with pathogens.

## 1. Introduction

Swine manure is a mixture of urine, feces, food residues, and water used in cleaning activities and contains a high load of microorganisms [1]. The manure microbial composition can vary depending on factors such as age, the type of animal, feed, manure dilution, and the storage technique [2], with a large bacterial population of saprophytic microorganisms, pathogenic bacteria, viruses, and fungi, as well as gastrointestinal parasite eggs and oocysts [3]. 

Of manure pathogens, special attention has been given to the enterobacteria group, as they have been pointed out as responsible for more than 2.2 million annual deaths caused by gastrointestinal problems [4]. Among enterobacteria, *E. coli* has been used as a fecal indicator for decades, but it can also be a pathogen due to its different strains, such as Enteroinvasive *E. coli* (EIEC), Enterotoxigenic *E. coli* (ETEC), Enteropathogenic *E. coli* (EPEC), Enterohemorrhagic *E. coli* (EHEC), Uropathogenic *E. coli* (UPEC), and Enteroaggregative *E. coli* (EaggEC) [5]. All around the world, diarrhea caused by pathogenic *E. coli* is responsible for 550 million diseases and 230,000 deaths each year [6]. Furthermore, prolonged oral exposure to these fecal contaminants has been linked to environmental enteropathy, a subclinical condition defined by chronic bowel inflammation that can contribute to structural changes in the small intestine and immune dysfunction in the patient [7]. Although the majority of *E. coli* types are innocuous, some variations are harmful to health and thus raise the risk of waterborne pathogens, such as *Salmonella* spp.

*Salmonella* spp. are rod-shaped, Gram-negative bacteria, with over 2500 serovars, that colonize the intestinal tract of animals and humans [8]. This bacteria has been reported by the World Health Organization (WHO) to be one of the antibiotic-resistant priority pathogens, requiring urgent strategies for infection management, including the reduction in this bacteria in environmental matrices [9].

Spreading manure on soil as a fertilizer is of special concern since it has been associated with environmental and public health issues due to the presence of zoonotic microorganisms, which can contaminate water [10] and may become associated with vegetable roots and be internalized [11]. For a long time, scientists considered that soil could act as a filter with the potential for self-purification, naturally reducing the pathogen load. However, studies have reported the migration of pathogens in soil, both vertically and horizontally, over a distance as far as 830 m [12]. This migration ability increases the possibility of water contamination [12]. 

Because of complex interactions among microorganisms and soil constituents, such as organic matter, and porosity, microbial transport across soils can differ [13]. Consequently, certain soil types are more susceptible to microbial migration [9,10]. In a study by Mantha et al. [13], *Salmonella enterica* leached more successfully through sandy soils than through organic soils. Furthermore, higher bacterial survival in organic soils and a rapid decrease in *Escherichia coli* (*E. coli*) concentrations in more nutrient-poor soil conditions have been reported [14,15,16,17,18]. When compared to sandy soils, which present non-cohesive particles and low organic matter retention, clayey soils offer greater water and nutrient retention capacities, ensuring bacterial survival [19,20]. Certain studies have shown this effect. For example, a study comparing *E. coli* O157:H7 survival after cattle slurry was applied to clayey and sandy soils found that survival in clayey soils could last up to 16 weeks compared to 8 weeks in sandy soil [21]. 

To facilitate the assimilation of manure or other liquid wastes into the soil matrix, agricultural soil is frequently tilled. Due to this practice, the size distribution of macropores changes, and the bulk density of the soil is temporarily reduced [22,23]. As a result, the soil has a considerable impact on the dynamics of pathogen transfer to groundwater sources. This necessitates a thorough understanding of pathogen movement and survival as they traverse the soil profile [24].

Currently, Brazil is the fourth-largest swine producer in the world, and the generated manure has been applied to soil as a fertilizer for many decades because it contains nutrients beneficial to plants and improves the soil structure [25]. The estimated volume of manure to be considered is determined from the daily volume excreted by the animal (8.6 L) for a herd of 38,212,374 animals [26,27]. However, in these regions, studies evaluating the survival of enterobacteria in soil are scarce. Therefore, we aimed to evaluate the survival, percolation, and leaching of enterobacteria in clayey soil after fertilization with swine manure. We spiked swine manure with *Salmonella enterica* Senftenberg (*S. senftenberg*) and *E. coli* and applied it to clayey soil. We then evaluated the survival, percolation, and leaching of the added enterobacteria.

## 2. Materials and Methods

### 2.1. Soil and Swine Manure Characterization

The soil and swine manure were sampled in the western region of Santa Catarina, Brazil. For the characterization, each sample of soil was dried in an oven (100 °C). The soil was then disaggregated with a mortar and pestle. All processes were carried out according to NBR 6457 [28]. The soil samples were classified by particle size using NBR 7181 [29], Atterberg’s limits (liquid limit—LL; plastic limit—PL) using NBR 7180 [30] and NBR 6459 [31], and the weight-specific grain value using ME 093 [32] (Table 1).

The total solid content was quantified using a gravimetric assay [33]. Total organic carbon was quantified using a TOC analyzer (Multi C/N 2100, Analytik Jena, Jena, Germany), at a flow rate of 160 mL min^−1^, using oxygen as a carrier. The temperature was set at 900 °C. Briefly, the samples were filtered through 0.45 µm membrane filters (Millipore, Burlington, MA, USA), acidified with phosphoric acid (40% w w ^−1^) (Sigma-Aldrich, EUA, St. Louis, MI, USA), and injected (250 µL) immediately into the analyzer. Calibration curves were generated by serial dilution of a stock solution of 1 g L^−1^ biphthalate (Synth, São Paulo, Brazil). 

Biological oxygen demand (BOD_5_) was determined in accordance with 5210-B, Standard Methods for the Examination of Water and Wastewater [33]. Alkalinity was determined by titration using sulfuric acid (0.1 M, Merck, Darmstadt, Germany) as a titrant. Alkalinity was determined as CaCO_3_ L^−1^: [(M × A × 10,000)/V]; where M is molarity of standardized acid (M); A is the acid volume dispensed to reduce sample pH to 4.5 (mL) and V is total sample volume (mL) [33]. 

The ascorbic acid colorimetric method was used to measure the concentration of phosphate-P (4500-P, Standard Procedures for the Analysis of Water and Wastewater [33]). The reagent solution was prepared using 50 mL of sulfuric acid (5 N) (Sigma-Aldrich, St. Louis, MI, USA), 5 mL of antimony potassium tartrate solution (Sigma-Aldrich, St. Louis, MI, USA), 15 mL of ammonium molybdate solution (Synth, São Paulo, Brazil), and 30 mL of ascorbic acid solution (Synth, São Paulo, Brazil). Subsequently, 0.8 mL of this solution was added to 5 mL of the previously filtered samples (0.45 μm membrane filter, Millipore, USA). After 10 min, the absorbance of each sample was measured in a UV-Visible spectrophotometer (Pharo 300, Merck) at 880 nm. The standard curves were generated by serially diluting a stock phosphate-P solution (0.05–0.2 mg-P L^−1^) (Merck, Darmstadt, Germany).

Potentiometric analysis using a selective electrode method was used to measure ammoniacal NH_3_-N (4500-NH_3_ D, Standard Procedures for the Analysis of Water and Wastewater [33]). The reagent solution was prepared NaOH/EDTA (10 N) (Neon, Sao Paulo, Brazil) and sodium hydroxide (10 N) (Neon, Sao Paulo, Brazil). The standard curves were generated by serially diluting a stock NH_3_-N solution (0.1–1000 mg-NH_3_-N L^−1^) (Merck, Darmstadt, Germany). The concentrations of nitrite-N and nitrate-N were determined by the N-(1-naphthyl)-ethylenediamine dihydrochloride colorimetric method and were measured at a wavelength of 550 nm (4500-NO_2_^-^B and 4500-NO_3_^-^F, Standard Procedures for the Analysis of Water and Wastewater [33]). Calibration curves were prepared by serial dilution of nitrite-N (0.1–2.0 mg-N L^−1^, Merck, Darmstadt, Germany) and nitrate-N (0.1–3.0 mg-N L^−1^, Merck, Darmstadt, Germany). pH was determined using a pHmeter (pH–mV, Hanna Instruments, Inc., Woonsocket, RI, USA). The data are shown in Table 2. 

### 2.2. Preparation of the Bacterial Inoculum

For the preparation of the inoculum spiked in swine manure, standard strains of *E. coli* and *S. enterica* serovar Senftemberg were spread on nutrient agar (Kasvi^®^) and incubated at 37 °C for 24 h. Following this, batches of bacterial colonies were gradually added to 10 mL of a 0.9% saline solution until they reached turbidity comparable to the 0.5 McFarland standard (Remel^®^), which contains 1.5 × 10^8^ bacteria per mL. This suspension was combined with swine manure and immediately applied to the soil. The volume of swine manure used in this study was comparable to that applied to corn, wheat, and soybean crops (50 m^3^ ha^−1^) [34].

### 2.3. Microbial Survival Assay

The sampled soil was deposited in 1 L reactors that were artificially contaminated with bacterial suspensions containing *E. coli* and *S. senftemberg* at concentrations comparable to the 0.5 McFarland standard (Remel^®^). Samples were collected at time zero (T0), daily, and every 5 days until all bacteria died. For *E. coli* quantification, samples were serially diluted at base 10, then placed at different depths in Chromocult^®^ Agar [35], and incubated at 37 °C for 24 h, and the count of typical colonies was determined according to the manufacturer’s instructions. To quantify *S. senftemberg*, the samples were serially diluted to base 10 in saline solution and placed on XLD Agar [36] for 24 h incubation at 37 °C, followed by standard colony counting according to the manufacturer’s instructions. The results are represented as colony-forming units (CFU). 

### 2.4. Microbial Percolation Assay 

Three soil column reactors, 70 cm high and 30 cm in diameter, fabricated in polyvinyl chloride tubes (PVC tubes) were used in the experiment. On the side, 1 cm diameter access slots were made at depths of 10, 20, 40, and 60 cm, to allow the soil sample collection during the experiment. 

The soils were rearranged in the columns in the same order in which they were removed from the original place on the farm (up to 60 cm deep). The columns were left alone for a week to allow the soil to stabilize [34]. Then, soils were fertilized with swine manure artificially contaminated with known concentrations of model bacteria. To monitor the percolation of microorganisms in the soil, 1 g soil samples were collected at different depths [34]. Samples were collected regularly until all bacteria died.

### 2.5. Microbial Leaching after Rain

To carry out the leaching experiments, after fertilization, the soil columns were exposed to a precipitation of 53 mm (at an environmental temperature of 20 °C). This experiment was conducted on a rainy day, representing the real conditions that occurring in the field. The rain volume was measured to calculate the precipitation. A tap was installed at the bottom to allow the leaching liquid to be collected. The leaching liquid from the soil was collected using a sterile collector tube, at times 2, 4, 8, 12, 24, 36, and 48 h after the rain and the enteric bacteria quantified [34].

### 2.6. Inactivation Kinetics

The inactivation coefficient and the time required for a 1 Log_10_ reduction of model bacteria (T_90_ = 1/−*k*) were calculated according to Ottoson et al. [37], considering the linear regression curve with r^2^ ≥ 0.75.

### 2.7. Statistical Analysis

*T*-test was used to evaluate the changes in enteric bacteria behavioral profiles in soil over time. One-way analysis of variance (ANOVA) was used to evaluate differences between the depths, using a 95% confidence level, followed by Bonferroni’s multiple comparison test (GraphPad Prism 5.0). The critical *p*-value for the test was set at ≤0.05.

## 3. Results and Discussion

### 3.1. Enterobacteriaceae Decay Profile in Soil

**Figure 1:**
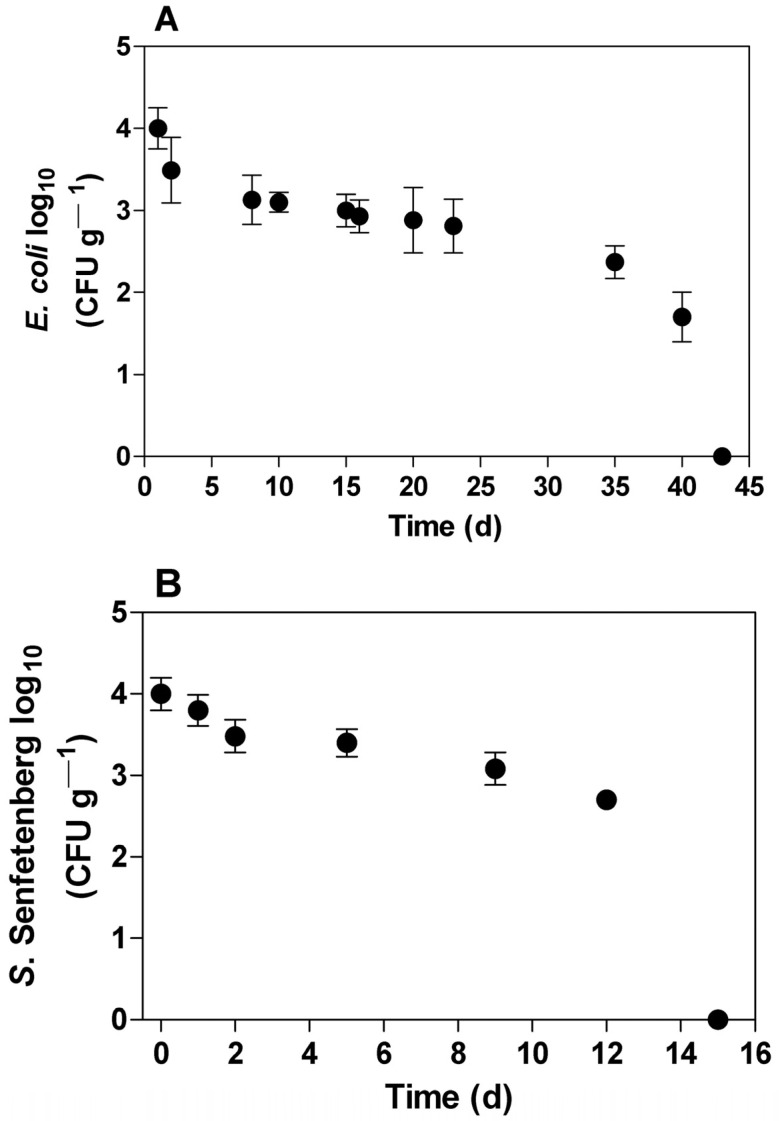
*E. coli* (**A**) and *S. senftenberg* (**B**) decay profiles in soil fertilized with swine manure over time.

The survival of pathogenic enterobacteria in clayey soil fertilized with swine manure spiked with *E. coli* and *S. senftemberg* is depicted in Figure 1. After 7 days, the *E. coli* concentration decreased by 90% (1 log_10_) and remained stable for 25 days; a significant decrease in *E. coli* concentration was observed after 43 days (*p* < 0.05). A different response was found for *S. senftemberg* (Figure 1B), where it required 9 days to reduce the concentration by 90% (1 log_10_). Additionally, 13 days were required for the elimination of *S. senftemberg* (10^4^ CFU). It is worth noting that untreated swine manure can present an *E. coli* concentration of 10^7^ MPN 100 mL^−1^ [38], so even after a 90% reduction, the bacteria load in manure remains high and is able to contaminate soil, where it can be active for more than 30 days. According to the World Health Organization, the recommendation for water reuse is an *E. coli* concentration lower than 10^3^ MPN mL^−1^ for the fertigation of cultures not directly consumed [39]. Previous studies conducted by our group showed that swine manure treatment consisting of anaerobic digestion followed by a pond system is suitable to remove pathogenic bacteria, leading to concentrations below 10^3^ CFU [40]. However, a low concentration of *E. coli* does not guarantee the absence of other pathogens, such as viral particles, which have environmental survival times greater than two months [41].

Pathogen survival in environmental matrices is affected by factors such as climatic conditions, temperature, pH, agrochemicals, aeration, soil type, and the presence of other microorganisms (due to predation or competition) [42,43]. Additionally, survival can be influenced by plants cultivated in the soil; Maule [44] reported that the greatest survival of bacteria occurs in soil containing rooted grass.

Similar results were observed after applying livestock manure to soil, where *E. coli* O157:H7 and *Salmonella* persisted in the soil for up to one month after its application to both sandy and clayey grassland soils [45]. Studies on the soil application of swine manure revealed that after the 20th day, the quantity of bacteria decreased very slowly, independent of the amount of sludge used, such that after 80 days, an estimated concentration of 10^3^ CFU dry matter^−1^ remained in the soil [46]. The estimated average time required to obtain undetectable *E. coli* concentrations in sandy soil ranged from 56 to 70 days [47].

*E. coli* O157:H7 continued to survive after 60 days in Brown soil sand and silts, with a decrease of 0.7 to 2.5 log_10_ CFU g^−1^. During the same period, the *E. coli* O157:H7 concentration in Brown soil clay containing natural organic matter increased by 0.58 log_10_ CFU g^−1^ compared to the original inoculation (from 6.68 to 7.26 log_10_ CFU g^−1^) [48]. On the other hand, the concentration of *E. coli* O157:H7 in Brown clay without natural organic matter had been reduced to undetectable levels by day 24 [48]. The clay concentration in soil has been recognized to have a significant impact on enterobacteria survival in soil, typically improving survival. Some of the most common clay minerals found in soils include kaolinite, montmorillonite, and illite [20]. Brennan et al. [20] studied the effect of clay mineral type on bacterial enterobacteria survival in soil. As a result, after 96 days of experimentation, the reduction in *E. coli* O157:H7 in the soil was 10^6^ CFU g, whereas, with the addition of kaolinite, montmorillonite, and illite, the reduction was 10^4^, 10^3^, and 10^2^ CFU g, respectively.

Clay minerals constitute the most active inorganic colloid components in soils, influencing bacterial adhesion, metabolism, colonization, and biofilm formation [49,50]. Clays with the highest surface areas and specific surface electrical characteristics were more efficient than silts and sands in attaching *E. coli* O157:H7 [48]. The attachment of bacteria, the first step in biofilm formation, stimulates the organism to produce extracellular polymeric substances such as polysaccharides, proteins, lipids, and nucleic acids, which form a protective matrix around the bacterial surface and protect cells from adverse environmental conditions [51]. In this respect, higher adhesion led to gradually longer *E. coli* O157:H7 survival in clay soil [48]. Surface-attached bacteria may have a different physiological or metabolic state in terms of gene transcription for growth and metabolism, which increases the chances of microbial species establishing and persisting in difficult environments [52].

### 3.2. Decay Kinetics of Enterobacteriaceae in Soil

Pathogens discharged with manure particles are exposed to various processes and routes that decide their die-off or growth, as well as their final deposition or fate [53]. Nevertheless, to contaminate water resources and possibly infect humans or animals, a pathogen must be able to survive after fertilization and endure the processes it may face at the soil surface, during transit through the soil, or after entrainment in the overland flow [54,55]. According to the findings in this study, *S. senftenberg* had a greater inactivation rate (0.096 d^−1^) compared to *E. coli* (0.1029 d^−1^) (Table 3). Additionally, for *E. coli*, a 90% reduction takes 9.71 days. *S. senftemberg* requires 10.4 days to be 90% inactivated (1 log_10_). Similar T_90_ values were obtained in sandy soils after swine digestate application for *S. enterica* Typhimurium (11.9 d) and *E. coli* O157:H7 (10.75 d) [34]. The inactivation coefficient (*k*) can be influenced by enterobacteria-specific and clayey mineral properties, as shown by Brennan et al. [20]. In this regard, *E. coli* O157:H7 exhibited *k* values of 0.30, 0.23, 0.15, and 0.06 in clayey soil (without mineral addition), a soil kaolinite mix, a soil illite mix, and a soil montmorillonite mix, respectively, whereas *Salmonella* Dublin exhibited *k* values of 0.30, 0.18, 0.20, and 0.05 in the clayey soil (without mineral addition), soil kaolinite mix, soil illite mix, and soil montmorillonite mix, respectively [20].

### 3.3. Percolation of Enterobacteriaceae in Soil

**Figure 2:**
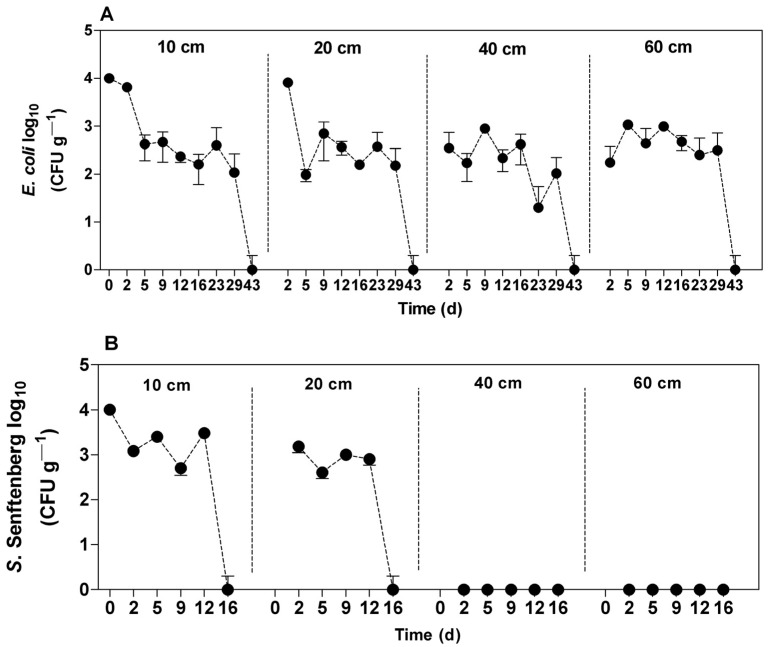
*E. coli* (**A**) and *S. senftenberg* (**B**) behavioral profiles in different soil depths fertilized with swine manure.

As shown in Figure 2A, *E. coli* was found up to a depth of 60 cm 48 h after swine manure application, most likely due to fertilizer drag. There was a significant reduction (*p* < 0.05) in the first five days at soil depths of 10 cm and 20 cm. *E. coli* strains remained viable in the soil column, similar to the survival results depicted in Figure 1A. *S. senftemberg* (Figure 2B) did not penetrate the deepest soil layers, reaching only a depth of 20 cm. There was a significant decrease in the *S. senftemberg* concentration in the layers of soil (10 and 20 cm) in the first 48 h and a reduction to zero by the 16th day after swine manure application (*p* < 0.05).

The movement of microorganisms in soil is influenced by intrinsic microbial features such as size, shape, cell surface characteristics, and biochemical and enzymatic properties [56]. In this sense, the differences observed between the bacteria used in this study could be explained by the cell size, where *Salmonella enterica* is a rod-shaped bacteria ranging from 2.2 to 5.0 μm [57], while *E. coli* cells are smaller at 1–2 μm [47,58], with smaller cells percolating longer. 

The number and size of microbial cells impact the settling velocity of manure. Microorganisms have a low density in general; hence, they are likely to remain suspended once entrained [54]. Suspended bacteria present in swine manure can travel quickly across the profiles of well-structured soils at moderate to high rates of water content through macropores and worm-holes. Any field soil that has macropores and receives enough water to fill these holes is likely to facilitate the fast transport of suspended bacteria to the depth at which these macropores are continuous [59]. A sandy soil with wider pores will allow for easier passage through the soil matrix than a clayey soil with fewer pore spaces [60]. Chemotactic migration permits motile bacteria to move more efficiently in response to environmental conditions (favorable or otherwise). They may also be capable of swimming toward soil pores and surface irregularities that would otherwise be inaccessible [60]; hence, their transport capability is increased. Others can use flagellar motion to move toward helpful substances such as nutrients, which promotes more mobility across the environmental medium [12].

Members of the *Pseudomonas*, *Achromobacter*, *Bacillus*, *Flavobacterium*, and *Enterobacter* genera have exhibited different transport potentials [61]. Sepehrnia et al. [17] reported that *E. coli* cells are expected to be more influenced by hydrodynamic forces compared to smaller-sized bacteria [17,62]. The adhesion of *Salmonella* to soil has been shown to be correlated with cell surface hydrophobicity [63]. Huysman and Verstraete [64] found that hydrophobic strains were 2–3 times slower to percolate through soil columns, as observed with the *Salmonella* in the present study.

### 3.4. Leaching of E. coli in Soil

**Figure 3:**
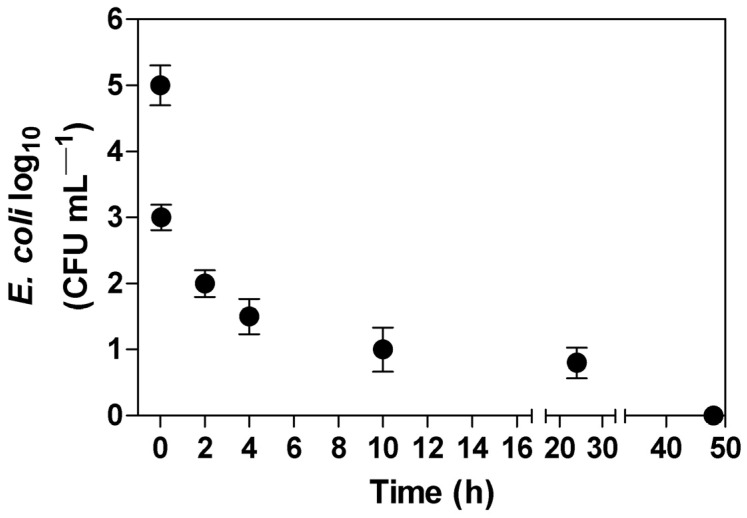
*E. coli* leaching profile in swine manure fertilized soil exposed to 53 mm rain.

Rain can promote the survival of pathogenic bacteria by keeping the soil wet, and it can also move bacteria through the soil to more or less suitable areas, as well as potentially contaminate groundwater [65]. Figure 3 shows the behavior of *E. coli* in clayey soil fertilized with swine manure exposed to rain. The samples obtained in this phase of the study were not from soil, but from the liquid fraction (leachate) that exceeded 60 cm of the soil column, simulating rains on swine-manure-fertilized soil. As a result, after 5 min of rain, approximately 10^3^ CFU reached a depth of 60 cm, and after 48 h, all water had percolated and the total bacteria concentration was reduced. This result indicates that the bacteria leaching in the first 24 h and the water eliminated in the last 24 h correspond to the water retained in the soil particles.

Furthermore, the use of liquid manure is predicted to improve microbial release and transport efficiency [66]. Manure compounds in liquid-based materials are more quickly recoverable and more influenced by the impact of precipitation or the flow of water than solid-manure compounds, which are more aggregated (adhered to material surfaces) [67,68]. Thus, since bacteria have greater mobility in the liquid phase than in the solid phase, liquid manure tends to be more uniformly polluted than solid manure [69].

Other studies reported the depth-dependent survival of *E. coli* and enterococci in soil following manure application and simulated rainfall of 30, 60, and 90 mm. In the first few days, *E. coli* concentrations increased and then gradually decreased to the initial amount; however, enterococci populations decreased at the beginning and were inactivated after 4 weeks, except when 30 mm of rain was applied: in this condition, the survival was longer than the 21 days of the experiment [70]. The bacterial activity decreases by one or two orders of magnitude for every 2 m of depth [71]. 

All of these findings highlight the diverse behavior of microorganisms in soil, depending on the soil type, microbial strains, manure load, and environmental conditions such as rain volume. During the application of manure without rain, there is a long survival period, but not with a long spread; in rainy periods, vertical leaching occurs faster. In this context, farmers should be encouraged to use environmentally friendly agriculture and manure management practices. Given the diversity of agricultural conditions, such farm and manure management solutions should be adaptable and pragmatic in design. A comprehensive combination of tactics that considers geographical, environmental, sociocultural, and economic differences would be suitable. Farmers’ knowledge and understanding must be improved, particularly in rural regions. It is critical to emphasize the need to use effective manure treatments and avoid applying new/raw manure [72].

## 4. Conclusions

This work evaluates the behavior (survival and percolation) of *E. coli* and *S. senftemberg* in clayey soils fertilized with swine manure. The results indicate that *E. coli* survives for a longer period (43 days) than *S. senftemberg* (14 days); *E. coli* percolates quickly through the soil. During a rainy event (53 mm), *E. coli* percolated 60 cm in less than 5 min, and it was possible to find viable bacteria up to 24 h after the rain. The results show the importance of reducing enteric pathogens in animal manures before their field application, which is critical for lowering the risk of produce-related foodborne diseases. Considering the characteristics of swine-producing regions, the load of effluents applied to the soil may exceed the self-purification capacity of the environment, and percolation or surface runoff may occur, with the consequent contamination of water bodies by pathogens.

## Figures and Tables

**Table 1:** Characterization of the natural clayey soil.

Soil particle size	Clay–Ø < 0.002 mm (%)	72.0
	Silt–0.002 mm < Ø < 0.06 mm (%)	22.5
	Thin sand–0.06 mm < Ø < 0.2 mm (%)	1.8
	Medium sand–0.2 mm < Ø < 0.6 mm (%)	2.2
	Coarse sand–0.6 mm < Ø < 2.0 mm (%)	1.5
	Boulder–2.0 mm < Ø < 60 mm (%)	0.0
Geotechnical characteristics	Liquid limit (%)	62
	Plastic limit (%)	37
	Plastic index (%)	25
	Specific weight of grains (g cm^−3^)	2.85
	Textural classification	Clay
	H.R.B *-A.A.S.H.T.O. **	A7-5

* Highway Research Board. ** American Association of State Highway and Transportation Officials.

**Table 2:** Physicochemical characteristics of swine manure used as a fertilizer.

Characteristic	Value
Total solids (g L^−1^)	3–8
Total organic carbon (g L^−1^)	1.5–6.5
BOD_5_ (g L^−1^)	2.5–4.5
Alkalinity as CaCO_3_ (g L^−1^)	5–8
Phosphate (g L^−1^)	0.048–0.07
Total nitrogen (g L^−1^)	1.5–2
Ammoniacal nitrogen (g L^−1^)	0.9–1.5
pH	7.9

**Table 3:** Inactivation coefficient (*k*), 90% reduction (T_90_) and correlation coefficient (r^2^) obtained from the linear model of the decay kinetics of *E. coli* and *S. senftenberg* in soil fertilized with swine manure.

Microorganism	*k* (d^−1^)	T_90_ (d)	r^2^
*E. coli*	0.1029	9.71	0.85
*S. senftenberg*	0.096	10.42	0.94

## Data Availability

Not applicable.

## References

[1] Lima T., Domingues S., Da Silva G.J. (2020). Manure as a Potential Hotspot for Antibiotic Resistance Dissemination by Horizontal Gene Transfer Events. Vet. Sci..

[2] Dadrasnia A., de Bona Muñoz I., Yáñez E.H., Lamkaddam I.U., Mora M., Ponsá S., Ahmed M., Argelaguet L.L., Williams P.M., Oatley-Radcliffe D.L. (2021). Sustainable Nutrient Recovery from Animal Manure: A Review of Current Best Practice Technology and the Potential for Freeze Concentration. J. Clean. Prod..

[3] Cotta M.A., Whitehead T.R., Zeltwanger R.L. (2003). Isolation, Characterization and Comparison of Bacteria from Swine Faeces and Manure Storage Pits. Environ. Microbiol..

[4] Ramírez-Castillo F., Loera-Muro A., Jacques M., Garneau P., Avelar-González F., Harel J., Guerrero-Barrera A. (2015). Waterborne Pathogens: Detection Methods and Challenges. Pathogens.

[5] Rossi E., Cimdins A., Lüthje P., Brauner A., Sjöling Å., Landini P., Römling U. (2018). “It’s a Gut Feeling”—*Escherichia coli* Biofilm Formation in the Gastrointestinal Tract Environment. Crit. Rev. Microbiol..

[6] Havelaar A.H., Kirk M.D., Torgerson P.R., Gibb H.J., Hald T., Lake R.J., Praet N., Bellinger D.C., de Silva N.R., Gargouri N. (2015). World Health Organization Global Estimates and Regional Comparisons of the Burden of Foodborne Disease in 2010. PLoS Med..

[7] Bass D., Stentiford G.D., Wang H.-C., Koskella B., Tyler C.R. (2019). The Pathobiome in Animal and Plant Diseases. Trends Ecol. Evol..

[8] Griffith R.W., Carlson S.A., Krull A.C. (2019). Salmonellosis. Diseases of Swine.

[9] Santos S., Martins C., Pereira C., Silvestre A., Rocha S. (2019). Current Challenges and Perspectives for the Use of Aqueous Plant Extracts in the Management of Bacterial Infections: The Case-Study of *Salmonella enterica* Serovars. Int. J. Mol. Sci..

[10] Ezugworie F.N., Igbokwe V.C., Onwosi C.O. (2021). Proliferation of Antibiotic-Resistant Microorganisms and Associated Genes during Composting: An Overview of the Potential Impacts on Public Health, Management and Future. Sci. Total Environ..

[11] Franz E., Visser A., Vandiepeningen A., Klerks M., Termorshuizen A., Vanbruggen A. (2007). Quantification of Contamination of Lettuce by GFP-Expressing *Escherichia coli* O157:H7 and *Salmonella enterica* Serovar Typhimurium. Food Microbiol..

[12] Abu-Ashour J., Joy D.M., Lee H., Whiteley H.R., Zelin S. (1994). Transport of Microorganisms through Soil. Water Air Soil Pollut..

[13] Mantha S., Anderson A., Acharya S.P., Harwood V.J., Weidhaas J. (2017). Transport and Attenuation of Salmonella Enterica, Fecal Indicator Bacteria and a Poultry Litter Marker Gene Are Correlated in Soil Columns. Sci. Total Environ..

[14] Villholth K.G., Jensen K.H., Fredericia J. (1998). Flow and Transport Processes in a Macroporous Subsurface-Drained Glacial till Soil I: Field Investigations. J. Hydrol..

[15] Horswell J., Hewitt J., Prosser J., Van Schaik A., Croucher D., Macdonald C., Burford P., Susarla P., Bickers P., Speir T. (2010). Mobility and Survival of *Salmonella* Typhimurium and Human Adenovirus from Spiked Sewage Sludge Applied to Soil Columns. J. Appl. Microbiol..

[16] Moradi A., Mosaddeghi M.R., Chavoshi E., Safadoust A., Soleimani M. (2019). Effect of Crude Oil-Induced Water Repellency on Transport of *Escherichia coli* and Bromide through Repacked and Physically-Weathered Soil Columns. Environ. Pollut..

[17] Sepehrnia N., Bachmann J., Hajabbasi M.A., Rezanezhad F., Lichner L., Hallett P.D., Coyne M. (2019). Transport, Retention, and Release of *Escherichia coli* and Rhodococcus Erythropolis through Dry Natural Soils as Affected by Water Repellency. Sci. Total Environ..

[18] Tate R.L. (1978). Cultural and Environmental Factors Affecting the Longevity of *Escherichia coli* in Histosols. Appl. Environ. Microbiol..

[19] Moynihan E.L., Richards K.G., Brennan F.P., Tyrrel S.F., Ritz K. (2015). Enteropathogen Survival in Soil from Different Land-Uses Is Predominantly Regulated by Microbial Community Composition. Appl. Soil Ecol..

[20] Brennan F.P., Moynihan E., Griffiths B.S., Hillier S., Owen J., Pendlowski H., Avery L.M. (2014). Clay Mineral Type Effect on Bacterial Enteropathogen Survival in Soil. Sci. Total Environ..

[21] Fenlon D.R., Ogden I.D., Vinten A., Svoboda I. (2000). The Fate of *Escherichia coli* and *E. coli* O157 in Cattle Slurry after Application to Land. J. Appl. Microbiol..

[22] Young I., Ritz K. (2000). Tillage, Habitat Space and Function of Soil Microbes. Soil Tillage Res..

[23] Shipitalo M.J., Gibbs F. (2000). Potential of Earthworm Burrows to Transmit Injected Animal Wastes to Tile Drains. Soil Sci. Soc. Am. J..

[24] Brennan F.P., O’Flaherty V., Kramers G., Grant J., Richards K.G. (2010). Long-Term Persistence and Leaching of *Escherichia coli* in Temperate Maritime Soils. Appl. Environ. Microbiol..

[25] ABPA (2021). ABPA—Associação Brasileira de Proteína Animal—Relatório Anual. São Paulo. Https://Abpa-Br.Org/Relatorios/.

[26] Oliveira P.A.V. (1993). Manual de Manejo e Utilizacão Dos Dejetos de Suínos.

[27] da Rosa G.A., Broetto L.F., Demczuk T., Viancelli A., Michelon W. (2022). Water Footprint and Productivity in Broilers and Swine Production in Brazil from 2008 to 2018. Environ. Sci. Pollut. Res..

[28] ABNT (2016). Amostra de Solo—Preparação Para Ensaios de Compactação e Ensaios de Caracterização.

[29] ABNT (2018). Solo—Análise Granulométrica.

[30] ABNT (2016). Solo—Determinação Do Limite de Plasticidade.

[31] ABNT (2017). Solo—Determinação Do Limite de Liquidez.

[32] DNER (1994). Solos—Determinação Da Densidade Real.

[33] APHA (2012). Standard Methods for the Examination for Water and Wastewater.

[34] Fongaro G., García-González M.C., Hernández M., Kunz A., Barardi C.R.M., Rodríguez-Lázaro D. (2017). Different Behavior of Enteric Bacteria and Viruses in Clay and Sandy Soils after Biofertilization with Swine Digestate. Front. Microbiol..

[35] Finney M., Smullen J., Foster H.A., Brokx S., Storey D.M. (2003). Evaluation of Chromocult Coliform Agar for the Detection and Enumeration of Enterobacteriaceae from Faecal Samples from Healthy Subjects. J. Microbiol. Methods.

[36] Magri M.E., Philippi L.S., Vinnerås B. (2013). Inactivation of Pathogens in Feces by Desiccation and Urea Treatment for Application in Urine-Diverting Dry Toilets. Appl. Environ. Microbiol..

[37] Ottoson J.R., Schnürer A., Vinnerås B. (2008). In Situ Ammonia Production as a Sanitation Agent during Anaerobic Digestion at Mesophilic Temperature. Lett. Appl. Microbiol..

[38] Moretti S.M.L., Bertoncini E.I., Abreu-Junior C.H. (2021). Characterization of Raw Swine Waste and Effluents Treated Anaerobically: Parameters for Brazilian Environmental Regulation Construction Aiming Agricultural Use. J. Mater. Cycles Waste Manag..

[39] Blumenthal U.J., Duncan Mara D., Peasey A., Ruiz-Palacios G., Stott R. (2000). Guidelines for the Microbiological Quality of Treated Wastewater Used in Agriculture: Recommendations for Revising WHO Guidelines. Bull. World Health Organ. Suppl..

[40] Viancelli A., Kunz A., Steinmetz R.L.R., Kich J.D., Souza C.K., Canal C.W., Coldebella A., Esteves P.A., Barardi C.R.M. (2013). Performance of Two Swine Manure Treatment Systems on Chemical Composition and on the Reduction of Pathogens. Chemosphere.

[41] Sánchez G., Bosch A. (2016). Survival of Enteric Viruses in the Environment and Food. Viruses in Foods.

[42] Alegbeleye O.O., Singleton I., Sant’Ana A.S. (2018). Sources and Contamination Routes of Microbial Pathogens to Fresh Produce during Field Cultivation: A Review. Food Microbiol..

[43] Mawdsley J.L., Bardgett R.D., Merry R.J., Pain B.F., Theodorou M.K. (1995). Pathogens in Livestock Waste, Their Potential for Movement through Soil and Environmental Pollution. Appl. Soil Ecol..

[44] Maule A. (2000). Survival of Verocytotoxigenic *Escherichia coli* O157 in Soil, Water and on Surfaces. J. Appl. Microbiol..

[45] Nicholson F.A., Groves S.J., Chambers B.J. (2005). Pathogen Survival during Livestock Manure Storage and Following Land Application. Bioresour. Technol..

[46] Estrada I.B., Aller A., Aller F., Gómez X., Morán A. (2004). The Survival of *Escherichia coli*, Faecal Coliforms and Enterobacteriaceae in General in Soil Treated with Sludge from Wastewater Treatment Plants. Bioresour. Technol..

[47] Côté C., Quessy S. (2005). Persistence of *Escherichia coli* and Salmonella in Surface Soil Following Application of Liquid Hog Manure for Production of Pickling Cucumbers. J. Food Prot..

[48] Liu X., Gao C., Ji D., Walker S.L., Huang Q., Cai P. (2017). Survival of *Escherichia coli* O157:H7 in Various Soil Particles: Importance of the Attached Bacterial Phenotype. Biol. Fertil. Soils.

[49] Cai P., Huang Q., Walker S.L. (2013). Deposition and Survival of *Escherichia coli* O157:H7 on Clay Minerals in a Parallel Plate Flow System. Environ. Sci. Technol..

[50] Huang Q., Wu H., Cai P., Fein J.B., Chen W. (2015). Atomic Force Microscopy Measurements of Bacterial Adhesion and Biofilm Formation onto Clay-Sized Particles. Sci. Rep..

[51] Busscher H.J., van der Mei H.C. (2012). How Do Bacteria Know They Are on a Surface and Regulate Their Response to an Adhering State?. PLoS Pathog..

[52] Donlan R.M., Costerton J.W. (2002). Biofilms: Survival Mechanisms of Clinically Relevant Microorganisms. Clin. Microbiol. Rev..

[53] Drummond J.D., Davies-Colley R.J., Stott R., Sukias J.P., Nagels J.W., Sharp A., Packman A.I. (2015). Microbial Transport, Retention, and Inactivation in Streams: A Combined Experimental and Stochastic Modeling Approach. Environ. Sci. Technol..

[54] Tyrrel S.F., Quinton J.N. (2003). Overland Flow Transport of Pathogens from Agricultural Land Receiving Faecal Wastes. J. Appl. Microbiol..

[55] Krog J.S., Forslund A., Larsen L.E., Dalsgaard A., Kjaer J., Olsen P., Schultz A.C. (2017). Leaching of Viruses and Other Microorganisms Naturally Occurring in Pig Slurry to Tile Drains on a Well-Structured Loamy Field in Denmark. Hydrogeol. J..

[56] Bernal M.P., Sommer S.G., Chadwick D., Qing C., Guoxue L., Michel F.C. (2017). Current Approaches and Future Trends in Compost Quality Criteria for Agronomic, Environmental, and Human Health Benefits. Advances in Agronomy.

[57] Ethelberg S., Mølbak K., Josefsen M.H. (2014). Bacteria: Salmonella Non-Typhi. Encyclopedia of Food Safety.

[58] El-Hajj Z.W., Newman E.B. (2015). How Much Territory Can a Single *E. coli* Cell Control?. Front. Microbiol..

[59] Smith M.S., Thomas G.W., White R.E., Ritonga D. (1985). Transport of *Escherichia coli* Through Intact and Disturbed Soil Columns. J. Environ. Qual..

[60] Jacobsen C.S., Bech T.B. (2012). Soil Survival of *Salmonella* and Transfer to Freshwater and Fresh Produce. Food Res. Int..

[61] Gannon J.T., Mingelgrin U., Alexander M., Wagenet R.J. (1991). Bacterial Transport through Homogeneous Soil. Soil Biol. Biochem..

[62] Wang Y., Bradford S.A., Šimůnek J. (2014). Estimation and Upscaling of Dual-Permeability Model Parameters for the Transport of *E. coli* D21g in Soils with Preferential Flow. J. Contam. Hydrol..

[63] Stenström T.A. (1989). Bacterial Hydrophobicity, an Overall Parameter for the Measurement of Adhesion Potential to Soil Particles. Appl. Environ. Microbiol..

[64] Huysman F., Verstraete W. (1993). Water-Facilitated Transport of Bacteria in Unsaturated Soil Columns: Influence of Cell Surface Hydrophobicity and Soil Properties. Soil Biol. Biochem..

[65] Saini R., Halverson L.J., Lorimor J.C. (2003). Rainfall Timing and Frequency Influence on Leaching of *Escherichia coli* RS2G through Soil Following Manure Application. J. Environ. Qual..

[66] Sepehrnia N., Memarianfard L., Moosavi A.A., Bachmann J., Guggenberger G., Rezanezhad F. (2017). Bacterial Mobilization and Transport through Manure Enriched Soils: Experiment and Modeling. J. Environ. Manage..

[67] Unc A., Goss M.J. (2004). Transport of Bacteria from Manure and Protection of Water Resources. Appl. Soil Ecol..

[68] Blaustein R.A., Pachepsky Y.A., Shelton D.R., Hill R.L. (2015). Release and Removal of Microorganisms from Land-Deposited Animal Waste and Animal Manures: A Review of Data and Models. J. Environ. Qual..

[69] Fangueiro D., Surgy S., Napier V., Menaia J., Vasconcelos E., Coutinho J. (2014). Impact of Slurry Management Strategies on Potential Leaching of Nutrients and Pathogens in a Sandy Soil Amended with Cattle Slurry. J. Environ. Manage..

[70] Stocker M.D., Pachepsky Y.A., Hill R.L., Shelton D.R. (2015). Depth-Dependent Survival of *Escherichia coli* and Enterococci in Soil after Manure Application and Simulated Rainfall. Appl. Environ. Microbiol..

[71] Fierer N., Schimel J.P., Holden P.A. (2003). Variations in Microbial Community Composition through Two Soil Depth Profiles. Soil Biol. Biochem..

[72] Alegbeleye O.O., Sant’Ana A.S. (2020). Manure-Borne Pathogens as an Important Source of Water Contamination: An Update on the Dynamics of Pathogen Survival/Transport as Well as Practical Risk Mitigation Strategies. Int. J. Hyg. Environ. Health.

